# *Enterocytozoon bieneusi* in European Domestic Ungulates and Pets: Occurrence, Genetic Diversity, and Public Health Perspectives from a Narrative Review

**DOI:** 10.3390/pathogens14111158

**Published:** 2025-11-13

**Authors:** Mirela Imre, Marius-Stelian Ilie, Tiana Florea, Corina Badea, Alexandra Pocinoc, Kálmán Imre

**Affiliations:** Faculty of Veterinary Medicine, University of Life Sciences “King Mihai I” from Timisoara, 300645 Timisoara, Romania; mariusilie@usvt.ro (M.-S.I.); corina.badea@usvt.ro (C.B.); kalmanimre@usvt.ro (K.I.)

**Keywords:** *E. bieneusi*, microsporidia, genotype diversity, zoonotic transmission, domestic ungulates, companion animals

## Abstract

*Enterocytozoon bieneusi* is the most frequently diagnosed microsporidian parasite in humans and a recognized cause of diarrheal disease, particularly in immunocompromised individuals. Its broad host range, which includes livestock, companion animals, and wildlife, highlights its zoonotic potential and warrants careful epidemiological assessment. This narrative review synthesizes available data on the occurrence and genetic diversity of *E. bieneusi* in European domestic ungulates (cattle, pigs, sheep, goats, horses, and water buffaloes) and pets (dogs and cats), aiming to provide an integrated perspective on animal reservoirs and their relevance for public health. Publications retrieved from the Web of Science Core Collection database were systematically screened, and country-specific results were extracted, emphasizing prevalence rates, genotype distributions, and zoonotic implications. Across Europe, cattle and pigs emerged as the most studied hosts, frequently harboring zoonotic group 1 genotypes such as I, J, BEB4, BEB6, and EbpA, while small ruminants, horses, and buffaloes remain comparatively undocumented. In pets, the dog-adapted genotype PtEb IX was predominant, but several zoonotic genotypes were also identified. Overall, the current evidence confirms the wide host range of *E. bieneusi* in Europe but also reveals significant data gaps compared to regions such as China, underlining the need for broader surveillance and harmonized molecular approaches within a One Health framework.

## 1. Introduction

Microsporidia are obligate intracellular spore-forming parasites that infect a wide range of vertebrate and invertebrate hosts [1,2]. They are characterized by a robust, chitin-containing spore wall (spores~1–4 µm) and a coiled polar filament that mediates host–cell invasion. Following ingestion, spores germinate and the polar filament rapidly everts to inject the infectious sporoplasm into intestinal epithelial cells, where intracellular replication proceeds through merogony (proliferative stages) and sporogony (sporont/sporoblast formation) culminating with the production of mature spores that are released into the intestinal lumen [1,2,3]. Inside enterocytes, the parasite can induce cytopathic changes, including microvillus blunting and epithelial damage, which contribute to malabsorption. Although primarily enteric, extra-intestinal tropisms (e.g., biliary tract, respiratory tissues) have been reported in immunocompromised hosts. The compact genome reduced metabolic repertoire, and intimate reliance on host-derived ATP and metabolites reflect extensive adaptation to an intracellular lifestyle and complicate in vitro culture and therapeutic targeting [1,2,3,4,5].

Despite the fact that they were once regarded as primitive protozoa, phylogenetic analyses have placed them within the fungal kingdom, in close association with the *Cryptomycota*. The group currently comprises more than 200 genera and over 1600 recognized species, with at least 17 species confirmed to infect humans. Among these, *Enterocytozoon bieneusi* is the most frequently detected, accounting for more than 90% of reported cases of human microsporidiosis [3,4].

The clinical relevance of *E. bieneusi* was first brought to light in 1985, following its detection in individuals suffering from human immunodeficiency virus (HIV) infection [2]. Since then, its role as an opportunistic pathogen has been well established, particularly in individuals with acquired immunodeficiency syndrome (AIDS), organ transplant recipients, cancer patients, the elderly, and young children [4]. In these groups, infection may cause persistent or life-threatening diarrhea, malabsorption, wasting, and other systemic complications [5]. Although self-limiting diarrhea can also occur in immunocompetent individuals, no fully effective treatment or vaccine is currently available, making prevention through control of transmission the most reliable approach [6].

Transmission occurs predominantly via the fecal–oral route. Infective spores, which are highly resistant in the environment, can contaminate water, food, and raw agricultural products, facilitating spread between animals and humans [4,6]. The parasite has been detected in a wide range of hosts, including livestock, companion animals, wildlife, and birds, underscoring its zoonotic potential [1]. The intensification of livestock production, particularly in cattle and pigs, raises concern about contamination of the environment and food chains, with potential spillovers into human populations.

Genetic characterization of *E. bieneusi* has relied mainly on the sequencing of the internal transcribed spacer (ITS) region of the rRNA gene. To date, more than 685 genotypes have been described worldwide. They are classified into 11–13 major phylogenetic groups [7,8]. Group 1 contains most zoonotic genotypes, including A, D, EbpC, and Type IV, which are commonly detected in humans. Group 2 has traditionally been considered ruminant-adapted, though some of its members (such as genotypes I, J, BEB4, and BEB6) have also been reported in humans, indicating that host specificity is not absolute. Groups 3–11 generally comprise host-adapted variants with limited or no known zoonotic significance [9,10].

To further emphasize the zoonotic importance of *E. bieneusi*, recent reports of human infections in Europe have raised increasing concern. Cases have been documented in several countries, including both immunocompromised and immunocompetent individuals (reviewed by [11]). These findings highlight that *E. bieneusi* is not only globally distributed but also an emerging public health issue throughout Europe.

Although global literature reports on *E. bieneusi* are extensive, knowledge from Europe remains fragmented. Several studies have addressed its presence in cattle, pigs, small ruminants, equids, dogs, and cats, but results vary considerably depending on host species, husbandry conditions, and molecular tools applied [10,11,12,13]. Notably, the repeated detection of zoonotic genotypes in domestic ungulates and companion animals points to their role as potential reservoirs for human infection. 

This narrative review summarizes the current knowledge on the occurrence and genetic diversity of *E. bieneusi* in European domestic ungulates and pets. By compiling and analyzing data from available studies, we highlight the distribution of genotypes across host species and countries, discuss their zoonotic potential, and outline the implications for public health within a One Health framework.

## 2. Classification and Nomenclature of *E. bieneusi* Genotypes

The classification of *E. bieneusi* genotypes has relied primarily on sequence analysis of the internal transcribed spacer (ITS) region of the ribosomal RNA gene, which provides a highly variable marker for differentiating strains. However, as molecular studies expanded rapidly during the early 2000s, inconsistent naming practices became a major obstacle. Identical ITS sequences were often assigned to multiple names in separate studies, leading to overlapping terminologies and confusion. For example, the widely reported zoonotic D genotype has been referred to under at least five alternative names, including PigITS9, PtEb VI, CEbC, and Peru9 [5,14]. Such redundancy complicated efforts to track host associations, geographic distributions, and zoonotic potential across studies.

In view of this challenge, a roundtable of international experts convened at the Tenth International Workshop on Opportunistic Pathogens (IWOP-10) and established a consensus system for genotype nomenclature. The central principle of this framework was that the first published name of a genotype has precedence and should be adopted in all subsequent studies. Later-assigned names should be treated as synonyms and reported alongside the primary designation to preserve continuity and assist readers in cross-referencing earlier literature. This approach balanced stability with inclusiveness, ensuring both clarity and historical traceability [14].

Applying this standard, Santín and Fayer [14] compiled all *E. bieneusi* ITS sequences available in GenBank^®^ at the time, tabulating genotype names, accession numbers, host species, and relevant publications. Their analysis identified 81 distinct genotypes carrying 111 different names. Of these, 26 genotypes were restricted to humans, eight occurred in both humans and other animals, 27 were found exclusively in livestock such as cattle and pigs, six were limited to companion animals, and 14 were associated with miscellaneous hosts. This broad distribution highlights both the genetic diversity of *E. bieneusi* and the risks of uncontrolled nomenclature proliferation.

Subsequent methodological advances have refined our understanding of *E. bieneusi* diversity. The development of a multilocus sequence typing (MLST) tool based on three microsatellites and one minisatellite loci demonstrated far greater discriminatory power than ITS alone, revealing substantial sub-structuring even within identical ITS genotypes [15,16]. Applications of MLST in both humans and animals further confirmed extensive cryptic diversity, emphasizing that ITS-based genotyping, while useful for broad classification, cannot always resolve epidemiologically relevant variation [16]. Recent reviews have reinforced these points, highlighting that while more than 800 ITS-defined genotypes are now recognized, multilocus or genomic approaches will be essential to improve resolution and to refine our understanding of host adaptation and zoonotic transmission [16,17].

The consensus nomenclature system, therefore, remains an important milestone in microsporidian epidemiology. By standardizing terminology, it enabled accurate comparisons between studies, facilitated meta-analyses, and improved assessments of host specificity and zoonotic risk. At the same time, its limitations underscore the need for complementary approaches such as MLST and whole-genome sequencing, which will likely provide the next generation of nomenclatural standards for this genetically diverse and epidemiologically important parasite.

## 3. Literature Search Strategy

A comprehensive literature search was conducted using the Web of Science Core Collection (WoSCC) database. This database was selected as the sole source of evidence because it is among the most authoritative and widely used bibliographic platforms for scholarly research, particularly in biomedical and life sciences. WoSCC was chosen over other databases for three main reasons:

(i) Breadth and quality of coverage—WoSCC indexes high-impact journals across disciplines, including parasitology, microbiology, veterinary medicine, and public health, ensuring broad coverage of studies relevant to *E. bieneusi*. Its rigorous inclusion criteria emphasize peer-reviewed, high-quality publications, minimizing the inclusion of non-scholarly or low-impact sources.
(ii) Advanced search functionalities—WoSCC allows refined query building using Boolean operators, proximity searching, and controlled indexing terms, facilitating a precise and reproducible search strategy. This ensures the retrieval of comprehensive yet targeted results, minimizing irrelevant literature.
(iii) Citation tracking and cross-referencing—WoSCC provides integrated citation analysis, enabling the identification of influential papers, highly cited reviews, and emerging trends within the field. This is particularly useful for a narrative review, where contextualizing research impact is essential.

Furthermore, the WoSCC was used as the primary database because of its comprehensive indexing of peer-reviewed journals in veterinary parasitology and zoonotic diseases, and its advanced, reproducible filtering capabilities. Only English-language full-text articles were considered. This approach ensured methodological consistency but introduced potential language and database-related bias. To assess the impact of this restriction, the search was cross-checked in PubMed and Scopus during manuscript revision. No additional eligible European studies matching the inclusion criteria were identified. Nevertheless, the reliance on a single database and exclusion of non-English literature remains a limitation, and future research could benefit from incorporating PubMed, Scopus, and regional repositories to enhance coverage and reduce selection bias.

The search strategy was structured around three sets of terms to ensure both specificity and comprehensiveness. The first set included the core keyword “*Enterocytozoon bieneusi*”, which directly targeted the parasite of interest. The second set incorporated the names of relevant host species, divided into domestic ungulates and companion animals. For the purposes of this review, the terms domestic ungulates included domesticated animals raised for agricultural purposes, specifically “cattle”, “pigs”, “sheep”, “goats”, “water buffalo” and “horses”, while the terms companion animals referred to pets commonly kept in households, specifically “dogs” and “cats”. These two groups were prioritized because of their high potential for direct or indirect contact with humans, and therefore greater public health relevance, compared to wild carnivores or wild ungulates, which have more limited opportunities for human exposure. The third set consisted of terms representing individual European countries, which was necessary because many studies report data at the country level rather than under the broader term “Europe.” By combining these three sets simultaneously, the strategy reduced irrelevant results while maximizing the likelihood of identifying all studies addressing the occurrence and genetic diversity of *E. bieneusi* in European domesticated ungulates and pets.

The search included studies published up to August 31, 2025. The final search string was: “*Enterocytozoon bieneusi*” AND (cattle OR pig OR sheep OR goat OR water buffalo OR horse OR dog OR cat) AND (Europe OR European countries OR “individual country name”). No restrictions on publication year were applied, but only studies published in English and indexed in peer-reviewed journals were considered. Initially, studies were selected through a systematic screening of titles and abstracts to identify publications potentially relevant to the objectives of this review. Articles that contained appropriate and pertinent information were then included for full-text assessment and subjected to a detailed, in-depth analysis to extract data on the occurrence, genotypes, and public health implications of *E. bieneusi* in European domesticated ungulates and companion animals. 

This review followed a narrative format but incorporated elements of systematic methodology. The search strategy, eligibility criteria, and data extraction steps were predefined before screening. A study selection flow chart summarizing identification, screening, eligibility and inclusion has been provided in Figure S1. Because of high heterogeneity in study design and reporting, a full PRISMA-compliant systematic review or meta-analysis could not be applied. Only peer-reviewed articles published in English with available full text were included. Conference papers, abstracts, theses, and non-English publications were excluded to ensure methodological transparency and data reliability. In studies investigating multiple host species, prevalence and genotype data were extracted separately for each animal species. To avoid duplication, datasets were carefully screened so that results from the same population or sample collection were not counted more than once across tables or analyses.

Although the WoSCC served as the sole bibliographic database for the structured search, reference lists of included articles were additionally screened to identify potentially relevant publications not indexed in WoSCC. Through this manual process, two studies from Turkey, one in cattle [18] and one in sheep [19] were identified and included, as they provided original molecular data on *E. bieneusi* in domestic ungulates. These exceptions were considered necessary to ensure completeness of the review. Finally, from a total of 140 articles retrieved through the initial search, 37 met the predefined inclusion criteria and were incorporated into the review. Several of these studies provided data on *E. bieneusi* screening across multiple host species.

## 4. Literature Findings

The literature sources comprise a broad spectrum of studies investigating *E. bieneusi* in European domestic ungulates and companion animals. Following the application of the inclusion criteria we identified publications covering cattle, pigs, sheep, goats, horses, and water buffaloes, as well as dogs and cats, all of which were retained for detailed assessment. Collectively, these studies provided insights into both the occurrence and molecular diversity of *E. bieneusi* across multiple host species and countries. 

Considerable variation was observed in study design, sample sizes, and diagnostic approaches, which contributed to differences in reported prevalence values. Despite this heterogeneity, certain patterns emerged: cattle and pigs were by far the most frequently investigated hosts, while goats and buffaloes remain comparatively understudied. In terms of genetic diversity, several genotypes were consistently dominant in livestock, with some variants showing strong zoonotic potential, being shared between humans and animals. Other genotypes appeared more host-adapted, pointing to a complex epidemiological landscape. To provide a clear overview of the available evidence, the findings of all studies included in this review are synthesized in Table 1 and Table 2, which summarize country-specific prevalence data, recorded genotypes, and their putative zoonotic relevance.

A formal meta-analysis was not performed due to substantial heterogeneity among studies in design, sampling strategy, host categories, diagnostic protocols, and incomplete reporting of confidence intervals. Instead, a pooled (weighted) prevalence was calculated per host species by dividing the total number of positive animals by the total number examined across studies (see Table 1 and Table 2). Genotype-specific pooled prevalence was not calculated as most publications reported genotype frequencies but not the total number of animals tested per genotype, making quantitative aggregation unreliable.

### 4.1. Domestic Ungulates

#### 4.1.1. Cattle

Among European domestic ungulates, cattle were the species most extensively studied for *E. bieneusi*. A total of 11 publications from Austria, the Czech Republic, Germany, Portugal, Slovakia, Spain, and Turkey reported the occurrence and genetic diversity of this parasite in bovine populations. According to Lichtmannsperger et al. [20], fecal samples from 351 cattle (calves and cows) yielded an overall prevalence of 4.6%. *E. bieneusi* was detected in both age groups, with the zoonotic genotype I and BEB6 being the most prevalent (Table 1). The authors emphasized the epidemiological importance of these genotypes, noting their wide distribution in ruminants and detection in humans. In the Czech Republic, Juránková et al. [21] investigated 432 animals from herds with and without bovine viral diarrhea virus (BVDV) infection and reported *E. bieneusi* in both groups, with an overall prevalence of 15.4%. Genotype I was detected, reinforcing its role as the main bovine-associated genotype in central Europe. Early European bovine cases were reported from Germany: Rinder et al. [22] identified *E. bieneusi* in cattle feces with a prevalence of 10.7% (3/28), while Dengjel et al. [23] subsequently identified five genotypes (EbpA, M, J, I and N) shared between cattle and humans, highlighting its zoonotic potential. These early studies demonstrated that cattle harbor zoonotic genotypes. In Portugal, Sulaiman et al. [24] studied 48 cattle and found *E. bieneusi* in 6.3% of animals. Genotyping revealed the presence of Type IV, as single genotype with zoonotic relevance. Likewise, Lobo et al. [25] documented bovine *E. bieneusi* infections as part of a broader survey in mammals. Although sample sizes were limited, the work confirmed the circulation of zoonotic genotypes (J and PtEb XI) in Portuguese cattle, consistent with findings from other European countries. In Slovakia, Valenčáková and Danišová [26] examined 100 cattle and detected *E. bieneusi* in 2 animals (2%), both of which were identified as genotype I, indicating a limited but zoonotically relevant occurrence in this population. Abarca et al. [27] examined cattle in northern Spain and identified the genotype BEB4. The presence of this genotype shared with humans confirmed the zoonotic potential of bovine isolates in the Iberian Peninsula. Another two independent studies highlighted the occurrence of *E. bieneusi* in Turkish cattle. Yildirim et al. [28] screened raw milk and found DNA of multiple genotypes (Table 1), some belonging to zoonotic group 1, and raised concerns about possible foodborne exposure through unpasteurized dairy products. In the same year, Bilgin et al. [18] reported a prevalence of 19.3% (29/150) in healthy cattle, identifying six genotypes: ERUSS1 (n = 24), N (n = 2), ERUSS2 (n = 1), ERUSS3 (n = 1), and ERUSS4 (n = 1). The predominance of ERUSS1, a genotype of uncertain zoonotic potential, suggests a largely host-adapted distribution, though the presence of other variants indicates some degree of genetic heterogeneity. Together, these studies confirm that both fecal and milk routes may be relevant for transmission.

Across Europe, cattle are a well-recognized reservoir of *E. bieneusi*, with prevalence values ranging from 2% to nearly 20% depending on herd type, age group, and diagnostic approach. Genotype I and BEB6 emerged as the dominant bovine-associated variants, repeatedly detected in central and western European countries and both reported from humans, underscoring their zoonotic significance. Additional genotypes such as BEB4, Type IV, J, and N, as well as country-specific variants (e.g., ERUSS1–4 in Turkey), highlight considerable genetic heterogeneity across regions. Importantly, several of these belong to group 1 genotypes with confirmed human relevance, while novel bovine-adapted genotypes also circulate. Together, the European evidence demonstrated that cattle not only sustain widespread and genetically diverse *E. bieneusi* populations but also contribute directly to the pool of zoonotic variants relevant for public health. 

**Table 1:** Summary of studies on *E. bieneusi* in European domesticated ungulates, with prevalence and genotype distribution. Genotypes in bold belongs to zoonotic groups.

Host	Country	Prevalence ^§^	Detected Genotype(s) ^†^ (n)	References
**Cattle (*Bos taurus*)**	Austria	4.6 (16/351)	**I ^a^** (12), **BEB4 ^b^** (3), **J ^c^** (2), **BEB8 ^d^** (1)	[20]
	Czech Republic	15.4 (37/240)	**I** (6),	[21]
	Germany	10.7 (3/28)	**I** (2), **J** (1)	[22]
		11.7 (7/60)	**EbpA ^e^** (1), **M** (1), **J** (3), **I** (1), **N** (1)	[23]
	Portugal	100 * (2/2)	**J** (1), **PtEb XI** (1)	[25]
		6.3 (3/48)	**Type IV ^f^** (3)	[24]
	Slovakia	2.0 (2/100)	**I** (2)	[26]
	Spain	0.6 (2/336)	**BEB4** (2)	[27]
	Turkey	4.5 **^#^** (9/200)	**ERUSS1** (5), **BEB6 ^g^** (3), TREb1 (1),	[28]
		19.3 (29/150)	ERUSS1 (24), **N** (2), ERUSS2 (1), ERUSS3 (1), ERUSS4 (1)	[18]
**Water buffaloes (*Bubalus bubalis*)**	Italy	13.5 (37/500)	**A** (2), **YNDCEB-90** (2), **I** (1)	[10]
	Turkey	2.7 (8/300)	YNDCEB-90 (5) and **J** (3)	[29]
		2.0 **^#^** (1/50)	TREb6 (1)	[28]
**Sheep (*Ovis aries*)**	Sweden	68.1 (49/72)	**BEB6** (40), **OEB1** (10), **OEB2** (6), ND (1) ^	[30]
	Portugal	2.2 (1/46)	**BEB6** (1)	[12]
	Turkey	18.0 **^#^** (36/200)	ERUSS1 (19), **BEB6** (11), TREb2 (2), TREb3 (2), TREb4 (1), TREb5 (1)	[28]
		8.0 (16/200)	**BEB6** (16)	[19]
**Goats (*Capra hircus*)**	Portugal	6.35 (4/63)	N.A.	[31]
	Spain	14.2 (1/7)	N.A.	[32]
**Pig (*Sus scrofa domesticus*)**	Czech Republic	93.7 (74/79)	**F ^e^** (70), **D ^h^** (2), **Peru9** (2)	[33]
	Germany	66.6 (4/6)	**F** (3), **G/H** (1)	[23]
		10.0 (5/50)	**F** (3), **G/H** (1), **O ^i^** (1)	[23]
		41.2 (14/34)	**O** (3), **E ^j^** (1), **F** (1), E1(1), F1(1), **E/F/G** (1), **H/F** (1)	[34]
	Spain	20.6 (7/34)	**I** (1)	[35]
		22.6 (42/186)	**EbpA**^e^ (22), **O** (8), **PigEb4** (3), **PigSpEb1** (3), **Pig HN-II** (2), **EbpA**+**PigEb4** (4)	[36]
	Slovakia	19.2 (5/26)	**F** (1), **I** (1), SVK-S1 (1), SVK-S2 (1), SVK-S3 (1)	[26]
	Switzerland	35.0 (38/109)	**F** (12), EbpB (6), **EbpC** (7), EbpD (3)	[37]
**Horses (*Equus caballus*)**	Czech Republic	17.5 (66/377)	**D** (34), **EpbA** (2), **G** (3), **WL15** (1) Horse 1 (7), Horse 2 (8) Horse 3 (2), Horse 4 (1), Horse 5 (1), Horse 6 (1), Horse 7 (1), Horse 8 (1), Horse 9 (1), Horse 10 (1), Horse 11 (2)	[38]
	Spain	0.0 (0/10)	-	[32]
	Turkey	18.7 (56/300)	ERUSS1 (24), **BEB6** (8), ERUH2 (6), ERUH3 (5), ERUH4 (4), ERUH5 (4), ERUH6 (3), ERUH7 (2)	[39]
	Switzerland	0.0 (0/24)	-	[37]

**^§^** Prevalence = number of positive animals/number of animals examined; values are presented as percentages; **^†^** Genotype names as reported in the original source; * Based on fecal samples pre-confirmed as *E. bieneusi* positive; **^#^** Prevalence derived from raw milk sample analysis; ^ Genotype undetermined; **^a^** Genotype I: also known as BEB2, CebE; **^b^** Genotype BEB4 synonym CHN1; ^c^ Genotype J: synonyms BEB1, PtEb X, CEbB; ^d^ Genotype BEB8 synonym CM19; ^e^ Genotype EbpA synonym F; **^f^** Genotype Type IV: also known as CMITS1, BEB5, BEB-var, K, Peru2, PtEbIII; **^g^** Genotype BEB6 synonym SH5; ^h^ Genotype D: synonyms PigEBITS9, WL8, Peru9, CEbC, PtEb VI; **^i^** Genotype O: also known as PigEBITS7, Peru 11; **^j^** Genotype E: synonyms EbpC, Peru4, WL13, CHG23, WL17.

#### 4.1.2. Water Buffaloes

Data on *E. bieneusi* in water buffaloes (*Bubalus bubalis*) in Europe remain scarce, but recent molecular studies from Italy and Turkey provide the first insights into its occurrence and genetic diversity in this species. Guadano-Procesi et al. [10] detected *E. bieneusi* in water buffaloe calves and confirmed its presence by sequencing and haplotype analysis. The study emphasized the detection of diverse genotypes with zoonotic potential (e.g., A, YNDCEB-90, I) within the *Bovidae* family and highlighted the epidemiological importance of buffaloes in the European context. Based on the obtained findings, the authors underlined the need for continued surveillance to clarify the zoonotic significance of the parasite in buffalo populations. Furthermore, Onder et al. [29] carried out a molecular survey in Turkish water buffaloes and documented *E. bieneusi* prevalence along with the identification of ITS genotypes. Their analysis revealed both host-adapted (YNDCEB-90) and zoonotic (I) variants. These findings confirmed that buffaloes in Turkey can harbor *E. bieneusi* genotypes of public health concern.

Together, these studies demonstrate that water buffaloes are susceptible to *E. bieneusi* infection and may serve as reservoirs of genetically diverse strains, including zoonotic ones. While current evidence remains limited to Italy and Turkey, the detection of group 1 genotypes and haplotype diversity underlines the need to incorporate buffaloes into broader epidemiological and One Health assessments of this parasite in Europe.

#### 4.1.3. Sheep

Reports on *E. bieneusi* in sheep across Europe are limited, but available studies demonstrate both the occurrence of infection and the circulation of genotypes with zoonotic relevance.

A Swedish survey of lambs reported that *E. bieneusi* was relatively common in this host species. Molecular analysis showed that genotype BEB6 was the dominant type, but also other genotypes with zoonotic potential (e.g., OEB1 and OEB2) have been recorded [30]. This genotype belongs to group 2, historically regarded as ruminant-adapted but also found in humans, thereby highlighting its zoonotic potential. The study emphasized that sheep could represent a relevant reservoir in northern Europe. More recently, *E. bieneusi* was identified in domestic sheep as part of a broader survey of wild and domestic animals in Portugal [12]. Molecular characterization revealed the presence of BEB6 genotype, well known for its zoonotic character. Although prevalence estimates were not the focus of the study, this finding reinforced the potential public health importance of sheep in Iberian contexts. In addition to studies targeting fecal samples, *E. bieneusi* DNA has also been detected in sheep milk in Turkey. Yildirim et al. [28] screened raw milk samples from cattle, sheep, and water buffaloes and identified several *E. bieneusi* genotypes in ovine milk. Importantly, some of these belonged to zoonotic group 1 (Table 1), suggesting possible foodborne transmission routes through unpasteurized dairy products. This study extended the epidemiological picture beyond direct fecal shedding, raising further concerns regarding exposure risk for consumers.

Overall, these reports demonstrate that sheep across different European regions can harbor *E. bieneusi*. The repeated detection of zoonotic genotype BEB6 in the available studies underscores the potential role of ovine populations in transmission cycles and highlights the need for their inclusion in One Health-oriented surveillance and control strategies.

#### 4.1.4. Goats

Evidence for *E. bieneusi* infection in goats in Europe remains limited, with only a few molecular studies reporting its occurrence. A recent survey of asymptomatic domestic ruminants conducted in northern Portugal demonstrated the presence of *E. bieneusi* in goats, confirmed by PCR and sequencing [31]. Phylogenetic analysis revealed that the isolates clustered within zoonotic groups, underscoring the potential role of goats as reservoirs of infection transmissible to humans. However, no genotype names or detailed genotyping results were provided. Nevertheless, the finding expanded the known host range of *E. bieneusi* in Portugal and underscored the public health relevance of caprine populations. In addition, an earlier study conducted in Galicia analyzed fecal samples from various domestic animals, including goats. Among the tested animals, *E. bieneusi* was identified in at least one goat, representing one of the first detections of this microsporidian parasite in caprine hosts in Europe [32]. While sample numbers were small, this finding contributed to the early recognition that goats can harbor *E. bieneusi* under natural farm conditions.

Taken together, these reports provide direct evidence that goats in the Iberian Peninsula are susceptible to *E. bieneusi* infection. Although the number of studies remains low, the identification of zoonotic genotypes in Portugal and the early detection in Spain support the notion that goats may represent an additional domestic reservoir relevant for public health.

#### 4.1.5. Pigs

Pigs are among the most frequently investigated livestock hosts for *Enterocytozoon bieneusi* in Europe, and numerous studies demonstrate both high infection rates and substantial genotype diversity (Table 1). Importantly, several of the genotypes identified in swine overlap with those detected in humans, emphasizing their zoonotic significance.

Sak et al. [33] reported the first detection of *E. bieneusi* on a Czech pig farm, documenting widespread occurrence and molecular confirmation of infection. ITS sequencing identified the overwhelming dominant occurrence of genotype F (synonym with EbpA), a well-documented type in humans, thereby raising concerns about swine acting as a reservoir for zoonotic transmission [33]. Several molecular studies have characterized *E. bieneusi* diversity in German pigs. Rinder et al. [22] provided early molecular evidence that pig and human *E. bieneusi* are closely related. In a follow-up study, Dengjel et al. [23] extended this finding by identifying genotype F in both pigs and humans, strongly supporting zoonotic potential. Later, Reetz et al. [34] extended the knowledge of parasite diversity by identifying two novel porcine genotypes, in addition to confirming infections with known types (Table 1). Collectively, these findings highlight the genetic heterogeneity of *E. bieneusi* in German swine and its clear zoonotic potential. Research in Spain has highlighted both clinical and epidemiological relevance. While Galván et al. [40] documented human microsporidiosis cases in transplant recipients and noted pigs as a potential source of infection with the genotype I, a later epidemiological study by Dashti et al. [36] demonstrated direct links between Iberian pigs and sympatric wild boars. The study revealed shared and novel *E. bieneusi* genotypes circulating across both populations, supporting the possibility of cross-species transmission at the wildlife–livestock interface. In Slovakia, a survey in pigs identified novel genotypes of *E. bieneusi*, expanding the known diversity of the parasite in central Europe. These new variants contributed to the growing recognition that swine harbor both zoonotic and host-adapted genotypes [26]. One of the earliest European studies of *E. bieneusi* in pigs was conducted in Switzerland and reported a high (about one-third of sampled swine) and diverse (4 distinct reported genotypes) prevalence). These genotypes differed from those found in humans at the time, leading the authors to suggest host specificity. However, later research in other countries has challenged this view by demonstrating overlap between porcine and human isolates [37]. 

Taken together, these reports confirm that pigs across Europe frequently harbor *E. bieneusi*, often at high prevalence. The repeated detection of human-associated genotypes such as F (EbpA) in swine from the Czech Republic, Germany or Spain emphasizes their zoonotic relevance. At the same time, the discovery of novel genotypes in Germany [34], Slovakia [26], and Spain [35] illustrates the substantial genetic diversity of swine isolates and indicates that pigs may act both as reservoirs of zoonotic genotypes and as hosts for lineages with more restricted host adaptation.

#### 4.1.6. Horses

Across Europe, molecular surveys have provided variable evidence for *E. bieneusi* in horses. While two studies (from the Czech Republic and Turkey) [38,39] confirmed equine infection with defined genotypes, earlier surveys in Spain [32] and Switzerland [37] reported no positives in the horses examined. 

Wagnerová et al. [38] examined 377 horses from 23 farms and reported *E. bieneusi* in 66 animals (17.3%). Fifteen ITS genotypes were identified, including six previously described types and nine novel variants. The zoonotic D genotype was the most frequent, accounting for over half of the positive samples. Other identified genotypes included EbpA, G, and WL15, which have also been detected in humans and various animals. The study highlighted management-related risk factors, with significantly higher prevalence among stabled horses compared to grazing horses and concluded that horses may serve as a potential reservoir for zoonotic transmission in this setting. Likewise, in Turkey, Yildirim et al. [38] conducted the first molecular prevalence study of *E. bieneusi* in Turkish horses, examining 300 animals from Central Anatolia. The parasite was detected in 18.7% (56/300). ITS sequencing revealed eight genotypes: two previously known (EbpC and BEB6) and six novel variants (designated ERUH2–ERUH7). Phylogenetic analysis grouped all sequences within ruminant-adapted cluster 2, suggesting that the genotypes identified in Turkish horses may have limited zoonotic potential [39]. Early surveys from Spain (Galicia) and Switzerland also included horses among the tested domestic animals, but none of the equine samples were positive for *E. bieneusi*, providing no evidence of infection in these populations [32,37].

Taken together, these European studies demonstrated that *E. bieneusi* occurred in horses, with prevalence rates ranging from 0% to nearly 19% depending on geographic region and study design. The detection of genotype D in Czech horses indicates zoonotic relevance, whereas the Turkish findings point to predominantly host-adapted genotypes with lower zoonotic potential.

### 4.2. Pets

#### 4.2.1. Dogs

An early European study detected *E. bieneusi* in farm dogs in Switzerland, identifying a single genotype (PtEb IX) by molecular characterization [41]. Although prevalence was low (8.3%) and sample size limited (n = 36), this investigation provided the first molecular evidence of canine infection in Europe. In Germany, Dengjel et al. [23] investigated 60 domestic dogs but found no *E. bieneusi*-positive samples, suggesting that dogs may play only a limited role in the parasite’s epidemiology in this country. In a recent shelter-based study, Szydłowicz et al. [42] reported a prevalence of 16.3% in dogs, with PtEb IX overwhelmingly dominant. This high prevalence in a confined shelter environment underlined the efficiency of dog-to-dog transmission, while the absence of zoonotic group 1 genotypes suggested limited immediate risk to humans in this cohort. Likewise, recently in Romania, Imre et al. [43] provided the first molecular evidence of *E. bieneusi* in dogs. Among diarrheic shelter animals, prevalence reached 9.8%, with two genotypes detected, including PtEb IX as dominant, and the zoonotic BEB4 variant, highlighting public health concerns in contexts of close dog–human contact. Lobo et al. [25] analyzed fecal specimens from dogs that had previously tested positive for *E. bieneusi*. Genotyping revealed the presence of both zoonotic variants, including genotype D and Peru6b, as well as the dog-adapted genotype PtEb IX. The detection of these mixed profiles indicates that while PtEb IX may circulate predominantly within canine populations, dogs in Portugal can also harbor genotypes of recognized zoonotic relevance. In Poland, two molecular surveys have assessed *E. bieneusi* in dogs. Piekarska et al. [44] reported a prevalence of 4.9% (4/82), identifying both the zoonotic genotype D (n = 2) and the dog-adapted PtEb IX (n = 2). More recently, Szydłowicz et al. [42] recorded a similar prevalence of 5.0%, again detecting PtEb IX (n = 3) together with genotype D. These consistent findings suggest that *E. bieneusi* occurs at low but stable levels in Polish dogs, with PtEb IX dominating the field but with zoonotic genotypes such as D circulating concurrently, thereby confirming a potential, albeit limited, public health relevance. In Spain, several studies have addressed canine populations. Early surveys detected *E. bieneusi* sporadically in dogs [32,45]. Later investigations provided more robust evidence: Galván-Díaz et al. [35] detected the genotype A, transmissible to humans, while Dashti et al. [46] reported a prevalence of 19.2% in northern Spain, with both PtEb IX and zoonotic genotype BEB6 identified. Together, these studies confirmed that Spanish dogs can harbor both host-adapted and zoonotic strains.

Altogether, the available European data indicate that dogs are susceptible to *E. bieneusi* but generally show lower prevalence compared to livestock (Table 2). The dog-adapted genotype PtEb IX was predominant across multiple regions, suggesting maintenance of infection within canine populations. However, the recurrent detection of zoonotic genotypes such as D, BEB4, BEB6, A, and Peru6b demonstrate that dogs can occasionally harbor strains of public-health concern. These findings underline the need for continued surveillance, particularly in shelters and multi-dog environments where transmission intensity appears highest.

**Table 2:** Overview of studies on *E. bieneusi* in cats and dogs within European countries, including prevalence, reported genotypes and host source. Genotypes highlighted in bold indicate those also described in humans.

Host	Country	Prevalence ^§^	Detected genotype(s) ^†^ (n)	Host Type	References
**Dogs (*Canis lupus familiaris*)**	Czech Republic	16.3 (14/86)	PtEb IX (14)	shelter	[42]
	Germany	0.0 (0/60)	-	N.A.	[23]
	Poland	4.9 (4/82)	**D ^a^** (2); PtEb IX (2)	household	[44]
		5.0 (5/101)	PtEb IX (3), **D** (1)	shelter	[42]
	Portugal	100 * (3/3)	**D** (1), **Peru6 ^b^** (1), PtEb IX (1)	household, shelter	[25]
	Romania	9.8 (11/112)	PtEb IX (10), **BEB4 ^c^** (1)	shelter	[43]
	Spain	0.8 (2/237)	**BEB6 ^d^** (1), PtEb IX (1)	owned	[46]
		19.2 (14/73)	**A ^e^** (7)	houshold	[35]
		11.7 (2/17)	N.A.	household	[32]
		8.7 (4/46)	N.A.	household	[45]
	Switzerland	8.3 (3/36)	PtEb IX (3)	farm	[41]
**Cats (*Felis catus*)**	Czech Republic	4.8 (3/63)	**D** (3)	stray	[47]
		0.0 (0/55)	-	pet	
	Germany	5.0 (3/60)	**K ^f^** (2), L (1)	N.A.	[23]
	Portugal	100 * (6/6)	**PtEbIII ^f^** (4), **PtEb IV** (1), PtEb VIII (1)	N.A.	[25]
	Poland	9.1 (4/44)	PtEb IX (3), Eb52 (1)	pet	[44]
		0.0 (0/31)	-	pet	[47]
		12.1 (4/33)	**D** (4)	stray	
	Turkey	5.6 (4/72)	**D** (2), **Type IV ^f^** (2)	pet	[48]
		50.1 (170/339)	**Type IV** (44), **D** (3),	stray	[49,50]
	Slovakia	0.0 (0/34)	-	pet	[47]
		12.8 (5/39)	**D** (5)	stray	
	Spain	3.0 (3/99)	**D** (2), **Peru11 ^g^** (1)	stray	[46]
		0.0 (0/10)	-	N.A.	[32]
		0.0 (0/9)	-	N.A.	[35]
	Switzerland	8.3 (1/12)	EbfelA (1)	farm	[41]

**^§^** Prevalence = number of positive animals/number of animals examined; values are presented as percentages; ^†^ Genotype names as reported in the original source; * Based on fecal samples pre-confirmed as *E. bieneusi* positive; N.A.—not available; **^a^** Genotype D: synonyms PigEBITS9, WL8, Peru9, CEbC, PtEbVI; **^b^** Genotype Peru6: also known as PtEb I, PtEbVII; **^c^** Genotype BEB4 synonym CHN1; **^d^** Genotype BEB6 synonym SH5; **^e^** Genotype A synonym Peru1; **^f^** Genotypes K, PtEbIII and Type IV: synonyms CMITS1, BEB5, BEB-var, Peru2; **^g^** Genotype Peru11 synonym Peru12.

#### 4.2.2. Cats

The first European feline case was documented by Mathis et al. [41], who identified *E. bieneusi* in a farm cat and characterized a novel genotype (EbfelA). Although based on a single positive sample, this study provided the earliest molecular evidence of feline infection in Europe. Two years later, Dengjel et al. [23] included 60 cats in their survey and reported a prevalence of 5.0%. Genotyping identified the zoonotic isolate belonging to genotype K and one non-zoonotic isolate of genotype L. These findings provided early molecular evidence of feline infection in Germany and highlighted that cats may carry both zoonotic and host-adapted *E. bieneusi* genotypes. In the Czech Republic, Kváč et al. [47] reported higher prevalence of *E. bieneusi* in stray cats (4.8%) compared to pet cats (0.0%). Molecular characterization revealed that strays were infected with the zoonotic D genotype, underlining their potential role in human exposure pathways. In the same multinational survey, Kváč et al. [47] also documented *E. bieneusi* in Slovak stray cats (Table 2). Again, the zoonotic D genotype was recorded, strengthening the view that free-roaming animals may act as important reservoirs. In Portugal, Lobo et al. [25] analyzed fecal specimens from cats previously confirmed as being *E. bieneusi* positive. Genotyping revealed zoonotic variants, including genotypes PtEb III and PtEb IV, beside the non-zoonotic PtEb VIII, underscoring the potential for both host-adapted and cross-species transmission. Two independent studies enriched the Turkish dataset. Pekmezci et al. [48] reported the first feline cases, identifying zoonotic genotypes in domestic cats (D and Type IV genotypes). Later, Erkunt Alak et al. [49] and Sürgeç et al. [50] conducted detailed prevalence and genotyping surveys of stray cats in İzmir, recording higher infection rates (50.1%) and documenting the dominance of the zoonotic Type IV genotype. Together, these results suggest that stray cats in Turkey represent a significant reservoir of zoonotic *E. bieneusi* genotypes. In Poland, a survey conducted in 2017 [44] identified *E. bieneusi* in cats with a relatively low prevalence (9.1%), recording non-zoonotic genotypes (Table 2). Complementary to this, in the same year, another investigation showed that stray cats were more frequently infected than pets (12.1% vs. 0.0%), with zoonotic genotype D, highlighting the potential epidemiological risk posed by free-roaming feline populations [47]. 

Several investigations have addressed feline populations in Spain. Early work by Lores et al. [32] included cats in their survey but found no evidence of *E. bieneusi* infection, suggesting limited involvement of this host in the parasite’s epidemiology in that country. Later, Galván-Díaz et al. [35] obtained the same results, while Dashti et al. [46] reported a prevalence of 3.0% in northern Spain, with D and Peru11 zoonotic genotypes. These findings confirm that Spanish cats can harbor variants with significance for public health.

Across the available European studies, cats are consistently shown to be susceptible to *E. bieneusi*, though prevalence varies widely by setting (Table 2). Stray cats generally display higher infection rates than pets and often carry zoonotic group 1 genotypes (including D, Type IV, and Peru11), raising public health concerns. At the same time, PtEb IX appears frequently, suggesting that a host-adapted cycle also exists within feline populations.

Although the present review focused primarily on animal hosts, environmental matrices such as surface water, wastewater and runoff are likely to play a complementary role in the European transmission cycle of *E. bieneusi.* Several studies from Europe reported the presence of ITS genotypes such as D, Type IV, BEB6 and EbpC in river waters, treated sewage, irrigation waters, and manure-contaminated environments, suggesting that spores shed by livestock and companion animals can enter aquatic systems and potentially reach humans (reviewed by [51,52]). These genotypes overlap with those detected in both humans and animals, supporting indirect, environmentally mediated zoonotic transmission. In contrast, data on soil or feed contamination are scarce or absent at molecular resolution. Therefore, environmental screening remains a gap in European surveillance and should be integrated into future One Health monitoring strategies.

### 4.3. Study Heterogeneity and Derived Limitations

The studies included in this review displayed considerable heterogeneity in design and methodology. Differences were observed in molecular diagnostic protocols, primer sets, sequencing success rates, and criteria used for genotype assignment. Sample matrices varied between feces, intestinal contents or raw milk which may influence detection sensitivity. In addition, host-related variability existed in terms of age categories (neonates, weaned animals, adults), health status, farming system and geographic origin. These inconsistencies hinder direct comparison of prevalence values and precluded stratified statistical analysis. Although this heterogeneity was acknowledged, limited reporting across studies did not allow systematic subgroup analysis. Future research would benefit from standardized sampling protocols, age-stratified data, harmonized PCR targets and sequencing techniques to improve comparability and enable formal meta-analytical approaches.

## 5. Conclusion Remarks and Future Perspectives

The evidence gathered in this review demonstrates that *E. bieneusi* is widely distributed among European domestic ungulates and companion animals, with marked differences in prevalence and genotype diversity between host species and regions. The literature indicates that pigs and cattle are the most extensively studied livestock hosts: they frequently yield zoonotic genotypes such as I, J, BEB4, BEB6, and EbpA, which were also reported in humans. Conversely, small ruminants, horses, and water buffaloes remain comparatively undocumented, despite their recognized importance in livestock production and their potential role in foodborne transmission. Other equids such as donkeys and mules were not included because no molecular data on *E. bieneusi* from these species were identified in our literature search focusing on Europe. This represents a gap that should be addressed in future research. Likewise, among pets, dogs and cats were confirmed hosts of both host-adapted and zoonotic variants, but available data remain limited to certain countries, often based on small sample sizes or isolated populations such as shelters or stray animals. To provide a simple summary measure, study-level prevalence data (number of positive animals/number examined) were pooled by host type across all included studies and expressed as weighted prevalence (total positives ÷ total animals examined). Using data from Table 1 and Table 2, the calculated weighted prevalence rates were: cattle 7.3% (110/1515), water buffalo 5.4% (46/850), sheep 19.7% (102/518), goats 7.1% (5/70), pigs 36.1% (189/524), horses 17.2% (122/711), dogs 7.1% (55/771) and cats 22.4% (203/906). These pooled figures are simple sample-size weighted summaries of the included studies and do not replace a formal meta-analysis. They should be interpreted cautiously given between-study heterogeneity (differences in sampling frame, diagnostic methods, and geographic sampling). Likewise, this review did not perform a full meta-analysis or produce forest plots because of the substantial heterogeneity in sampling strategies, diagnostic protocols and reporting formats among studies. Instead, pooled weighted prevalence values were calculated to provide a quantitative overview by host species. Future European studies that standardize sampling and molecular typing would allow meta-analytic models and forest plot–based comparisons.

Several ITS genotypes reported in European animals have also been identified in human clinical isolates. Notably, genotypes D, Type IV, BEB6, BEB4, I, and EbpA, commonly detected in cattle, pigs, horses, dogs, and cats, have been documented in human cases in multiple European countries, including Czech Republic, France, Germany, Poland, Portugal, Russia, Slovakia, and Spain (reviewed by [11]). These genotypes belong primarily to zoonotic Group 1 and Group 2 lineages, confirming cross-species circulation. This overlap supports the hypothesis that livestock and companion animals may act as reservoirs for human infection, particularly in areas with close human–animal contact or environmental contamination.

When contrasted with the global literature, particularly from China where large-scale molecular surveys have been conducted across a wide array of hosts [7,17], European data appear fragmented and insufficiently comprehensive. Surveillance gaps remain particularly evident in underrepresented regions (e.g., Scandinavia, the Balkans, Baltic states) and in wildlife and environmental reservoirs. Greater harmonization of molecular workflows and coordinated One Health surveillance would significantly improve data comparability, trend analysis and risk assessment across Europe. The limited number of eligible European studies reflects a true gap in published molecular data rather than a narrow search strategy, highlighting the need for more standardized and geographically comprehensive research. This gap hampers our ability to fully evaluate the epidemiological role of European animal populations, especially in relation to zoonotic spillovers and regional genotype distribution. Furthermore, the repeated detection of zoonotic genotypes in animals closely associated with humans underscores the need to strengthen *One Health* surveillance frameworks that integrate veterinary, medical, and environmental data. 

Future research should prioritize several areas. First, systematic molecular surveys covering underrepresented host species and geographic regions are essential to establish a more complete picture of *E. bieneusi* circulation in Europe. Second, greater use of multilocus sequence typing and whole-genome sequencing is needed to refine our understanding of intra-genotypic diversity, host adaptation, and transmission dynamics beyond the resolution offered by ITS typing alone. Third, longitudinal studies addressing transmission routes, particularly the role of milk, water, and shared environments, would clarify exposure risks for humans. Finally, harmonization of diagnostic protocols and genotype nomenclature remains vital to ensure comparability of results and enable robust meta-analyses.

Overall, while European studies provide valuable insights, the current evidence base is not yet sufficient to match the depth of knowledge generated in other regions. Addressing these gaps will be critical for assessing zoonotic risks, guiding control measures, and integrating *E. bieneusi* more effectively into public health and veterinary monitoring strategies.

## Data Availability

No new data were created or analyzed in this study.

## Supplementary Materials

The following supporting information can be downloaded at: https://www.mdpi.com/article/10.3390/pathogens14111158/s1.
