# Supplementary material for: Enterocytozoon bieneusi in European Domestic Ungulates and Pets: Occurrence, Genetic Diversity, and Public Health Perspectives from a Narrative Review
Source: Pathogens. 2025 Nov 13;14(11):1158. doi: 10.3390/pathogens14111158 (PMC12655100; doi:10.3390/pathogens14111158)
Supplement: Supplementary file 1 [file pathogens-14-01158-s001.zip › pathogens-3918567-supplementary.pdf]

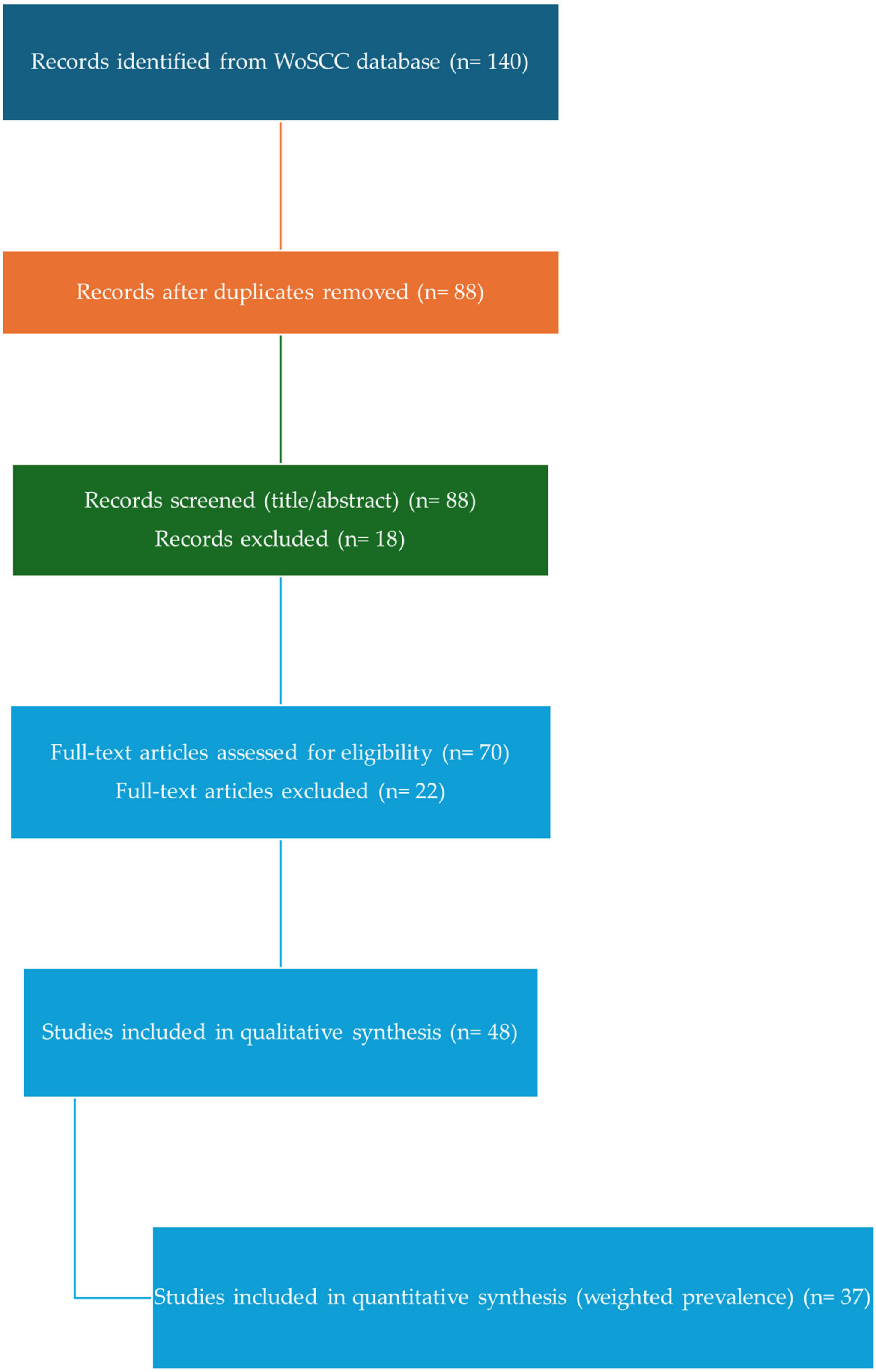

Figure S1. Simplified PRISMA-ScR flow diagram illustrating the process of study identification, screening, eligibility assessment, and inclusion for the review of *E. bieneusi* in European domestic ungulates and companion animals
